# Analytical Method Development of Benzisothiazolinone, a Biocide, Using LC–MS/MS and a Pharmacokinetic Application in Rat Biological Matrices

**DOI:** 10.3390/molecules28020845

**Published:** 2023-01-14

**Authors:** Seong Jun Jo, Zhouchi Huang, Chae Bin Lee, Soon Uk Chae, Soo Hyeon Bae, Soo Kyung Bae

**Affiliations:** 1 Integrated Research Institute of Pharmaceutical Sciences, College of Pharmacy, The Catholic University of Korea, Bucheon 14662, Republic of Korea; 2 Q-Fitter, Inc., Seoul 06578, Republic of Korea

**Keywords:** benzisothiazolinone, rat, biological matrices, LC–MS/MS, intravenous, dermal application

## Abstract

Benzisothiazolinone (BIT), a biocide widely used as a preservative in household cleaning and personal care products, is cytotoxic to lung cells and a known skin allergen in humans, which highlights the importance of assessing its toxicity and pharmacokinetics. In this study, a simple, sensitive, and accurate LC–MS/MS method for the quantification of BIT in rat plasma, urine, or tissue homogenates (50 μL) using phenacetin as an internal standard was developed and validated. Samples were extracted with ethyl acetate and separated using a Kinetex phenyl–hexyl column (100 × 2.1 mm, 2.6 μm) with isocratic 0.1% formic acid in methanol and distilled water over a run time of 6 min. Positive electrospray ionization with multiple reaction monitoring transitions of *m*/*z* 152.2 > 134.1 for BIT and 180.2 > 110.1 for phenacetin was used for quantification. This assay achieved good linearity in the calibration ranges of 2–2000 ng/mL (plasma and urine) and 10–1000 ng/mL (tissue homogenates), with *r* ≥ 0.9929. All validation parameters met the acceptance criteria. BIT pharmacokinetics was evaluated via an intravenous and dermal application. This is the first study that evaluated BIT pharmacokinetics in rats, providing insights into the relationship between BIT exposure and toxicity and a basis for future risk assessment studies in humans.

## 1. Introduction

Common consumer products, such as household cleaning and personal care products, are easily contaminated by microorganisms. Biocides, which can inhibit microbial growth, are incorporated into these products to avoid consumer health risks that result from bacterial contamination. Isothiazolinones, which are isothiazole-based heterocyclic chemical compounds, are known for their significant antifungal and antibacterial properties. Isothiazolinone representative compounds include methylchloroisothiazolinone (CMIT), methylisothiazolinone (MIT), kathon (a 3:1 mixture of CMIT and MIT), benzisothiazolinone (BIT), octylisothiazolinone (OIT), and dichloro-octylthiazolinone (DCOIT) [1]. BIT is widely used as a preservative in household cleaning and personal care products, such as paints and varnishes, polishing agents, pesticides, disposable gloves, nail polishes, liquid hand soaps, and sunscreens [2,3,4]. As these products are extensively used, humans are being systematically exposed to BIT. Human exposure occurs mainly through skin contact or inhalation rather than orally [5]. MIT, CMIT, and BIT are known to cause allergic contact dermatitis [6], with BIT being recognized as an allergen since 1976 [7]. Furthermore, citral/BIT mixtures used as additives in air fresheners considerably decrease the viability of lung epithelial cells and inhibit cell proliferation in a dose-dependent manner [8]. Kwak et al. [9] also reported that when inhaled, BIT can increase mucus production and upregulate MUC5AC expression in the airway, which may be associated with mucus hypersecretion related to airway inflammatory diseases.

Although the EU and Switzerland prohibited the use of isothiazolinones in leave-on cosmetics owing to their toxicity, thus banning BIT-containing cosmetics, BIT use in cosmetics is still permitted in the US and Canada [10].

Taken together, these studies indicate that continuous exposure to isothiazolinones through various products can be harmful to human health. However, to precisely assess isothiazolinone risks, it is necessary to understand their toxicokinetics in the body via various exposure routes. To appropriately interpret the relationship(s) between exposure and toxicity (or safety) data, a precise and sensitive quantification method is required. To the best of our knowledge, no analytical method has been reported for measuring BIT in biological samples. A report on a validated analytical method of MIT in plasma and the pharmacokinetic profile of MIT in rats was published [11], while several analytical methods for measuring BIT in consumer products or environmental samples, such as water, were reported [12,13,14,15,16,17,18]. However, most methods cannot be directly used to assay BIT in biological fluids because of the interference from endogenous materials and the lack of sensitivity or the lack of full validation. Therefore, a simple and sensitive analytical method for the determination of BIT in biological samples must be developed.

Given this background, the aims of this study were as follows: (1) Develop and validate a simple and sensitive LC–MS/MS method for the quantification of BIT in rat plasma; urine; and various organs, namely, the brain, liver, lung, heart, kidney, and spleen. (2) Explore the pharmacokinetic properties of BIT in rats administered through an intravenous injection or dermal application, which was the main exposure route using the LC–MS/MS method developed. The results of the present study could be the basis for attaining a more mechanistic understanding of BIT exposure and aid future BIT risk assessment studies in humans.

## 2. Results and Discussion

### 2.1. Optimization of Chromatographic Conditions and MS Detections

**Figure 1:**
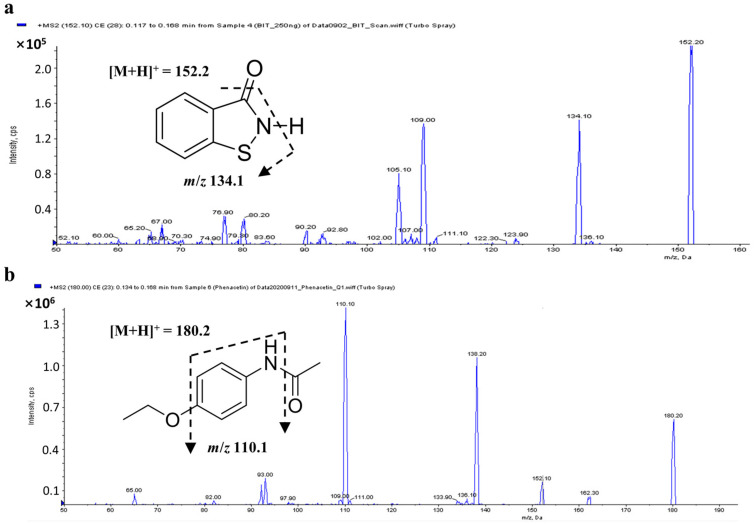
Chemical structures and product ion mass spectra of (**a**) benzisothiazolinone and (**b**) phenacetin, with [M + H]^+^ at *m*/*z* 152.2 and 180.2 as the precursor ions.

In this study, a sensitive and precise LC–MS/MS method was developed to quantify BIT in rat biological matrices, such as plasma, urine, and tissues. Ion scan products of BIT and the internal standard (IS; phenacetin) were obtained in scan mode after individual standard solutions were injected into the mass spectrometer. Positive-mode electrospray ionization (ESI) yielded a better spectrometric response for BIT and the IS than negative-mode ESI. Therefore, [M + H]^+^ ions at *m*/*z* 152.2 for BIT and *m*/*z* 180.2 for the the IS were used in MS/MS mode to obtain fragmentation patterns (Figure 1). In BIT, several product ions with different abundances were observed in the full-scan product ion mass spectra, including *m*/*z* 105.1, 109.0, and 134.1 (Figure 1). In the transition of *m*/*z* 152.2 > 109.0, an interfering endogenous peak close to the retention time of BIT was detected. Thus, we used another abundant ion of BIT (*m*/*z* = 134.1) as the quantifier ion, while *m*/*z* 109.0 and 105.1 were used as the qualifier ions. For the IS, the most intense product ion at *m*/*z* 110.1 was used for the quantification, and the less intense product ion at *m*/*z* 138.2 was used as the qualifier ion (Figure 1). Mass spectrometric parameters were optimized based on the maximal response of the selected product ions.

The chromatographic conditions were optimized to achieve the efficient separation of unknown substances present in rat plasma and urine by using different columns (including C18, C8, and phenyl–hexyl columns) and various mobile phase compositions. The phenyl–hexyl column with an isocratic mobile phase comprising 0.1% formic acid in distilled water and 0.1% formic acid in methanol (75:25, *v/v*) resulted in the best symmetrical peak shape and the highest sensitivity and was selected for further analysis. Initially, sample preparation was attempted using methanol and acetonitrile for protein precipitation, resulting in significant matrix effects and lower sensitivity. Thus, a liquid–liquid extraction procedure was evaluated using several organic solvents, including ethyl acetate, ether, dichloromethane, acetone, chloroform, methyl *tert*-butyl ether, and their mixtures. Ethyl acetate was found to be optimal, resulting in a clean chromatogram for the blank plasma samples, consistent extraction recovery, and negligible matrix effects.

It is highly desirable to select a suitable IS with an appropriate retention, extraction recovery, and degree of matrix effect as an analyte in LC–MS/MS analysis. Following these criteria, a stable isotope of the analyte would be considered a good IS. However, as a commercially stable isotope of BIT is not available, several compounds were investigated for the selection of a suitable IS, and finally, phenacetin was found to be the most appropriate.

### 2.2. Method Validation

#### 2.2.1. Specificity

**Figure 2:**
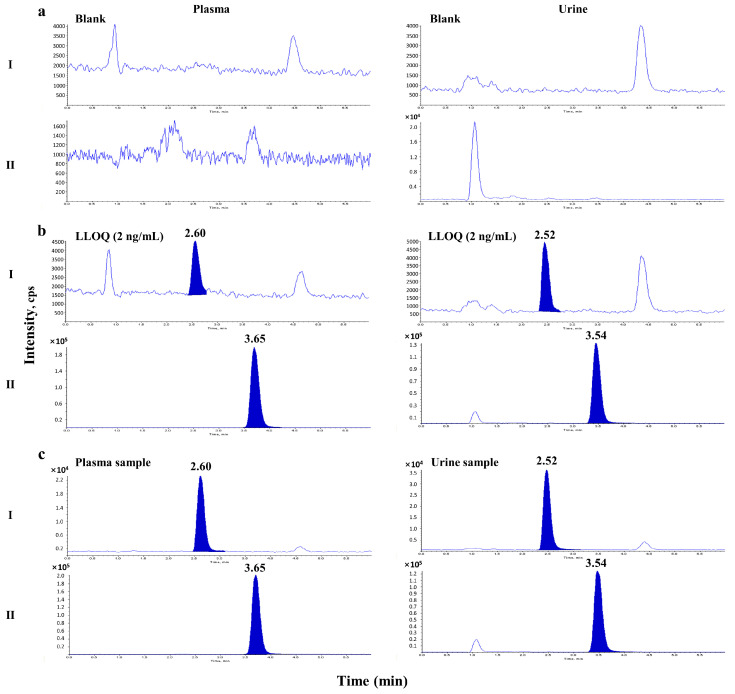
Representative chromatograms of benzisothiazolinone (BIT) (**I**) and phenacetin (IS) (**II**) in plasma and urine: (**a**) double blank plasma or urine; (**b**) blank plasma or urine spiked with BIT at the LLOQ (2 ng/mL for plasma and urine) and the IS; (**c**) a plasma sample from 90 min after intravenous injection of 10 mg/kg BIT and a urine sample collected from 0 to 24 h after intravenous injection of 10 mg/kg BIT.

**Figure 3:**
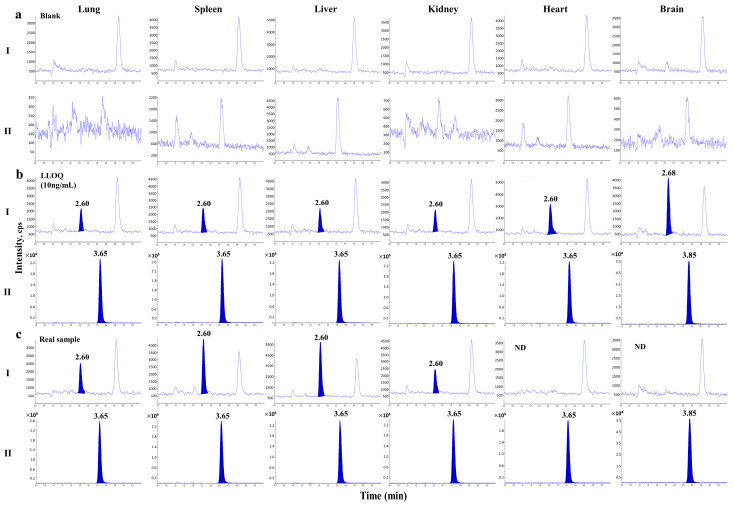
Representative chromatograms of BIT (**I**) and phenacetin (IS) (**II**) in six rat homogenates (lung, spleen, liver, kidney, heart, and brain): (**a**) double blank tissue homogenates; (**b**) blank tissue homogenates spiked with BIT at the LLOQ (10 ng/mL) and the IS; (**c**) real samples for lung and spleen from 180 min after intravenous administration of 10 mg/kg BIT and real samples for liver and kidney from 30 min after intravenous administration of 10 mg/kg BIT. ND, not detected.

No obvious endogenous interference from the six batches of rat biological samples (plasma, urine, and six different tissue homogenates) was observed at the elution times of BIT (2.60 min) and the IS (3.65 min). Figure 2 shows representative LC–MS/MS chromatograms of blank plasma or urine (Figure 2a), plasma and urine samples at the lower limit of quantification (LLOQ; 2 ng/mL) and the IS (Figure 2b), and a plasma sample from 90 min and a urine sample collected from 0 to 24 h after intravenous injection of 10 mg/kg BIT (Figure 2c). The total run time for each sample was 6 min. Representative LC–MS/MS chromatograms of the blank tissue homogenates and LLOQ samples are shown in Figure 3.

#### 2.2.2. Linearity and Sensitivity

The concentration–response relationship was fitted using a 1/x^2^ weighted regression model to evaluate the linearity of the calibration curves for BIT in rat plasma (2–2000 ng/mL), urine (2–2000 ng/mL), and six tissue homogenates (10–1000 ng/mL for each). Correlation coefficients r ≥ 0.9929 were obtained for all the calibration curves used in this validation assay. The equations of the calibration curve for rat plasma and urine were y = 0.00530x + 0.000205 and y = 0.00604x + 0.00105, respectively, where y is the BIT/IS peak area ratio and x is the BIT concentration. The back-calculated results for all the calibration standards were within ±15% of the nominal value. The LLOQ in plasma, urine, and the six tissue homogenates were 2, 2, and 10 ng/mL, respectively, with acceptable accuracy and precision (within ±20%). The LLOQ was sufficient for this method to be used for evaluating the pharmacokinetic properties of BIT in rats.

#### 2.2.3. Precision and Accuracy

The intra- and inter-day precision and accuracy values for BIT in rat plasma and urine samples are summarized in Table 1. All accuracy results were within ±15% of the nominal value and all precision results were <15%, validating the accuracy and precision of the developed method. The precision and accuracy data for six rat tissue homogenates (brain, liver, lung, kidney, heart, and spleen) are presented in Table 2 and were within the acceptable range of ±15%.

#### 2.2.4. Dilution Integrity

After a 100-fold dilution of six aliquots of the BIT plasma sample (150 μg/mL) with blank plasma, the accuracy and precision (relative standard deviation (RSD)) were estimated to be 101% and 6.55%, respectively, satisfying the acceptance criteria [17]. This indicated that plasma samples with concentrations exceeding the upper limit of quantification (up to 75-fold) could be measured reliably after appropriate dilution.

#### 2.2.5. Extraction Recovery and Matrix Effects

The mean extraction recovery of BIT in plasma samples (urine) at the three quality control (QC) levels ranged from 90.8% to 93.5% (90.4–92.7%), with RSDs of 6.56–8.00% (1.83–2.93%), and the matrix effects ranged from 102% to 108% (97.8–101%), with RSDs of 3.31–6.11% (3.56–6.27%) (Table 1). The extraction recovery of the IS and matrix effects were 99.1 ± 4.54% and 102 ± 3.50%, respectively (Table 1). Thus, by having low matrix effects and a highly reproducible recovery, this assay was shown to be reliable for bioanalysis. The mean extraction recovery and matrix effects of BIT in the six tissue homogenates are summarized in Table 2.

#### 2.2.6. Stability

The BIT stability data in rat plasma and urine are summarized in Table 3. BIT in rat plasma and urine was found to be stable under various typical storage and processing conditions during routine sample analysis. Both the precision and accuracy were within the acceptable limit of 15% for all tested QC samples. Stock solutions of BIT were also found to be stable for 30 days after storage at 4 °C and 90 days at −80 °C. Under these storage conditions, the BIT concentration was found to be more than 99.2% of the freshly prepared samples.

### 2.3. Application of the Developed Method for BIT Pharmacokinetic Studies in Rats

The novel validated LC–MS/MS method developed in this study was successfully employed to examine the pharmacokinetics and tissue distribution of BIT following intravenous and dermal administration.

#### 2.3.1. Intravenous Injection of BIT in Rats

**Figure 4:**
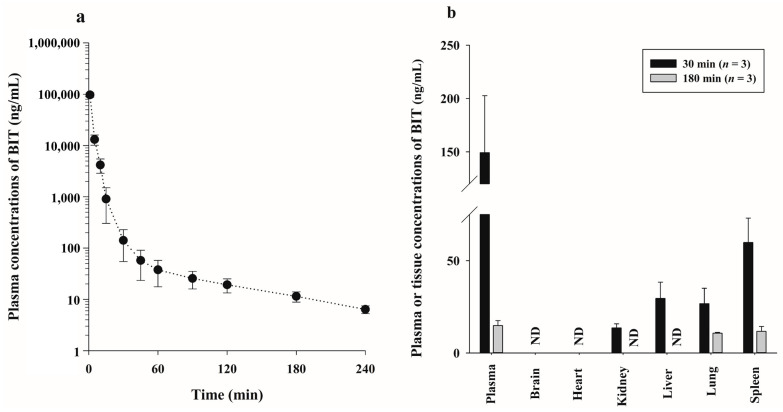
(**a**) Mean plasma concentration–time profiles of benzisothiazolinone (BIT) after an intravenous injection of 10 mg/kg into rats (*n* = 6); (**b**) mean concentration of BIT in various tissues at 30 min (*n* = 3) and 180 min (*n* = 3) after the intravenous injection. Vertical bars represent the SD. ND, not detected.

BIT was intravenously injected into rats (*n* = 6) at a dose of 10 mg/kg. The plasma concentration–time profiles of BIT are shown in Figure 4a, and the relevant pharmacokinetic parameters are listed in Table 4. BIT was removed rapidly from the systemic circulation, with a terminal half-life (t_1/2_) value of 79.2 ± 10.4 min and a total body clearance (CL) value of 28.3 ± 3.79 mL/min/kg. The volume of distribution in a steady state (Vd_ss_) was relatively small at 129 ± 19.0 mL/kg, indicating that BIT distribution in tissues was limited. The percentage of unmetabolized BIT excreted in urine over the course of 24 h was almost negligible. The Ae_0–24 h_ value was 0.0506 ± 0.0136%, suggesting that the majority (>99.95%) of BIT was eliminated via a non-renal (mainly hepatic) route. The percentage of unmetabolized BIT recovered from the gastrointestinal tract, including its contents and feces at 24 h (GI_24 h_), was 0.0324 ± 0.00639%, indicating that BIT was excreted into the bile and/or secreted into the gastrointestinal tract, although the proportion was very small (Table 4).

#### 2.3.2. Tissue Distribution after Intravenous Injection of BIT

Figure 4b shows the quantitative distribution of BIT in tissues at 30 min (*n* = 3) and 180 min (*n* = 3) after a 10 mg/kg intravenous injection in the rat brain, heart, kidney, liver, lung, and spleen. At 30 min, although it was detected in the liver, lung, kidney, and spleen homogenates, the corresponding BIT concentrations were lower than those in the plasma (Figure 4b). This was evident in the calculated tissue-to-plasma ratios in the liver, lung, kidney, and spleen at 30 min, which were <1.0, with values of 0.221 ± 0.0940, 0.197 ± 0.100, 0.100 ± 0.0380, and 0.423 ± 0.102, respectively. The tissue concentration of BIT at 180 min was much lower than that at 30 min (Figure 4b). BIT was not detected in the brain, liver, heart, or kidney, whereas trace amounts were found in the lungs and spleen. The tissue-to-plasma ratios of the lungs and spleen at 180 min were 0.740 ± 0.156 and 0.830 ± 0.347, respectively. These data indicated that the rat organs studied had a low affinity for BIT, which was supported by the considerably small Vd_ss_ value of BIT (129 ± 19.0 mL/kg) (Table 4).

#### 2.3.3. Dermal Application of BIT on Rats

**Figure 5:**
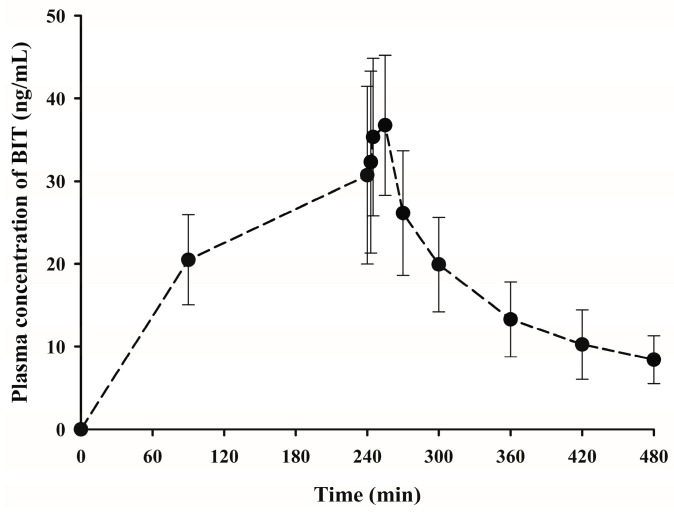
Mean plasma concentration–time profiles of benzisothiazolinone after a dermal application of 10 mg on rats (*n* = 8). Vertical bars represent the SD.

Dermal absorption is a more common exposure route for personal care products and household cleaning agents than both ingestion and intravascular injection [2,3,4,5]. Therefore, the in vivo percutaneous absorption of BIT in this study was examined in rats after the dermal application of a lotion formulation. BIT was dermally applied in a 7 × 10 cm^2^ area of shaved dorsal skin at a dose of 10 mg/rat (*n* = 8). The application area was then covered for 4 h using a dressing and bandage. The plasma concentration–time profiles of BIT following the dermal application are shown in Figure 5, and the corresponding pharmacokinetic parameters are presented in Table 5. The plasma concentration of BIT increased steadily as long as the bandage was attached, indicating that BIT was continuously absorbed through the skin. After reaching T_max_, the plasma concentrations of BIT slowly declined, with a t_1/2_ value of 101 ± 15.7 min (Figure 5, Table 5). After 4 h of dermal application, the remaining and/or washed amount detected at the application site was subtracted from the initial dermal application amount to calculate the amount absorbed into the systemic circulation. Approximately 7.20% (0.720 ± 0.122 mg/rat) of the dermally applied dose penetrated the dorsal skin (~2.67 mg/kg based on body weight). Thus, the *F* value of BIT following dermal application in rats was calculated to be 11.0% (Table 5).

## 3. Materials and Methods

### 3.1. Materials and Reagents

BIT (CAS no. 2634-33-35, purity 97.8%; Figure 1), dimethyl sulfoxide, formic acid, lanolin (CAS no. 8006-54-0), petrolatum (CAS no. 8009-03-8), stearic acid (CAS no. 57-11-4, purity 98.5%), propylparaben (CAS no. 94-13-3), methylparaben (CAS no. 99-76-3), disodium edetate (CAS no. 6381-92-6), propylene glycol (CAS no. 57-55-6), triethanolamine (CAS no. 102-71-6, purity 99.0%), and phenacetin (CAS no. 62-44-2, IS, purity ≥ 98%; Figure 1) were purchased from Sigma-Aldrich (St. Louis, MO, USA), while homosalate (CAS no. 118-56-9) was purchased from the Tokyo Chemical Industry (Tokyo, Japan). HPLC-grade methanol, ethyl alcohol, and ethyl acetate were obtained from Burdick and Jackson Co. (Morristown, NJ, USA). Deionized water was obtained using a Milli-Q Plus Ultrapure Water System (Millipore, Bedford, MA, USA).

### 3.2. Animals

Male and female Sprague Dawley rats (8 to 10 weeks old, 250–310 g) were purchased from Orient Bio (Seongnam, Gyeonggi-do, Republic of Korea). The protocol of this animal study was approved by the Department of Laboratory Animals, Institutional Animal Care and Use Committee on the Songsim Campus of The Catholic University of Korea (approval no. 2019-022). The rats were housed under controlled environmental conditions (20 ± 2 °C, 55 ± 5% relative humidity, 12 h light/dark cycle). The animals were acclimated for 1 week, with free access to water and food before their use for pharmacokinetic studies. Male rats were used for the intravenous injection study, including the tissue distribution study (*n* = 12) and female rats were used for the dermal application study (*n* = 8).

### 3.3. LC–MS/MS Conditions

Rat plasma, urine, and tissue samples containing BIT were analyzed using a 1260 HPLC system (Agilent Technologies, Wilmington, DE, USA) coupled with a tandem mass spectrometer QTRAP 5500 (AB SCIEX, Foster City, CA, USA) equipped with an ESI interface operated in positive ion mode. Chromatographic separation was performed using a Kinetex phenyl–hexyl column (100 × 2.1 mm, 2.6 μm, Phenomenex, Torrance, CA, USA) with an isocratic mobile phase comprising 0.1% formic acid in methanol and 0.1% formic acid in distilled water (25:75, *v*/*v*) at a flow rate of 0.4 mL/min. The column and autosampler temperatures were maintained at 40 and 4 °C, respectively. The injection volume was 2 μL, and the total run time was 6.0 min per sample. A unit mass resolution was used, and the dwell time was set to 150 ms. Quantification was performed in multiple reaction monitoring modes as follows: BIT at *m*/*z* 152.2 > 134.1 (quantitation) and 109.0 and 105.1 (confirmation); IS at *m*/*z* 180.2 > 110.1 (quantitation) and 138.2 (confirmation). The parameters for mass spectrometry were set as follows: 5500 V ion spray voltage; 500 °C ion source temperature; 30 and 20 psi for ion source gases 1 and 2, respectively; 20 psi curtain gas; 50 V for BIT and 70 V for IS declustering potential (DP); 10 V entrance potential (EP); 12 V collision cell exit potential (CXP); 28 eV for BIT and 25 eV for IS collision energy. The analytical data were processed using Analyst software (version 1.5.2; Applied Biosystems, Foster City, CA, USA).

### 3.4. Stock Solutions, Calibration Standards, and Quality Controls

Stock solutions of BIT and IS (1 mg/mL) were prepared in methanol and stored at −80 °C. The IS stock solution was diluted to 10 ng/mL using methanol for routine use. A series of working solutions for BIT was prepared by diluting the stock solution with methanol to obtain the desired concentrations. All working solutions were stored at 4 °C and transferred to room temperature (25 °C) prior to use.

Calibration curves were prepared by spiking 49 μL of drug-free rat plasma or urine with 1 μL of the appropriate working solution to yield a series of concentrations (2, 5, 10, 50, 200, 1000, and 2000 ng/mL). Rat plasma or urine quality control (QC) samples were separately prepared to acquire four different concentrations of 2, 4, 400, and 1600 ng/mL, corresponding to the lower limit of quantification (LLOQ), low QC, medium QC, and high QC, respectively.

The calibration standards in the rat tissue homogenates were prepared by spiking 49 μL of drug-free rat tissue homogenate with 1 μL of the appropriate working solutions to yield a series of concentrations (10, 20, 50, 200, and 1000 ng/mL). QC samples were prepared using the same procedure as described above (30, 150, and 800 ng/mL). Drug-free rat tissue homogenates were prepared by homogenizing the rat tissues with appropriate volumes (tissue weight/solution volume, 1:2, *w/v*, g/mL) of 0.9% injectable NaCl solution using a tissue homogenizer (Ultra-Turrax T25, Janke and Kunkel IKA-Labortechnik, Staufen, Germany). All calibration standards and QC samples were freshly prepared before the analysis.

### 3.5. Sample Preparation

An aliquot of 50 μL of rat plasma, urine, or tissue homogenates and 10 μL of IS solution containing 10 ng/mL phenacetin were added to a 1.5 mL tube, and the mixture was extracted with 1 mL ethyl acetate by vortexing for 10 min. After centrifugation at 18,000× *g* for 5 min at 4 °C, 1 mL of the upper layer was transferred to a clean tube and evaporated to dryness under a gentle stream of nitrogen at 40 °C. The residue was reconstituted with 80 μL of methanol and centrifuged (18,000× *g* for 5 min at 4 °C). Then, a 2 μL aliquot was injected into the LC–MS/MS system for analysis. Rat tissue samples (0.5 g) or the entire sample (<0.5 g) were homogenized in 0.9% injectable NaCl solution at 1:2 (*w/v*); then, 50 μL of tissue homogenate was processed using a method similar to that used for the plasma and urine sample preparation.

### 3.6. Method Validation

The validation parameters assessed were specificity, linearity, precision, accuracy, recovery, matrix effects, dilution integrity, and stability of BIT in rat plasma, urine, and various tissues (liver, lung, heart, kidney, and spleen) in accordance with the international bioanalytical method validation industry guidelines [19,20].

#### 3.6.1. Specificity

The specificity of the method was evaluated by analyzing rat plasma, urine, or tissue samples from at least six different lots to investigate potential interferences at the LC peak region corresponding to BIT and the IS. The specificity acceptance criterion was that at least four of six lots should have a ≤20% area response compared with that of the LLOQ-level response (2 ng/mL for plasma and urine, 10 ng/mL for the six tissue homogenates) in the same matrix.

#### 3.6.2. Linearity and Sensitivity

Calibration curves were constructed by using standard BIT concentrations ranging from 2 to 2000 ng/mL for plasma and urine and 10–1000 ng/mL for six rat tissue samples (brain, liver, lung, heart, kidney, and spleen). The linearity of each method-matched calibration curve was determined by plotting the peak area ratio (y) of BIT to the IS versus the nominal BIT concentration (x). The calibration model was selected based on linear regression analysis with different weighting factors (1/x, 1/x^2^, and none). The calibration curves had a correlation coefficient (r) of 0.99 or better. The acceptance criteria for each back-calculated concentration should satisfy the accuracy criterion. The LLOQ was defined as the lowest analyte concentration on the calibration curve with precision (±20%) and accuracy (80–120%), which should be at least five times that of the blank response.

#### 3.6.3. Precision and Accuracy

The precision and accuracy were assessed by analyzing six replicates of an LLOQ sample (2 ng/mL for plasma and urine, 10 ng/mL for the six tissues) and three different QC samples (4, 400, and 1600 ng/mL for plasma and urine; 30, 150, and 800 ng/mL for the six tissues) on the same day (intra-day) and another 10 replicates for five consecutive days (inter-day). The actual concentration of each QC level, including that of the LLOQ sample, was calculated using a calibration curve constructed on the same day. Precision was expressed as the percent relative standard deviation (%RSD) and accuracy as (observed concentration)/(spiked concentration) × 100%. The acceptance criterion was within a ±15% deviation from the normal value, except in the case of the LLOQ, which was ±20%.

#### 3.6.4. Dilution Integrity

A dilution integrity test was performed to ensure that the final concentration of plasma samples was not affected by dilution with the blank plasma. Six replicates of BIT-spiked rat plasma samples (150 μg/mL) were diluted 100-fold to the calibration range with blank plasma, processed, and analyzed with undiluted calibration standards and accuracy and precision values within acceptable limits.

#### 3.6.5. Extraction Recovery and Matrix Effects

Extraction recovery was determined by comparing the mean peak areas of six replicates obtained from the extracted spiked sample with those of the post-extracted spiked samples at three QC levels (4, 400, and 1600 ng/mL for plasma and urine; 30, 150, and 800 ng/mL for the six tissues). The matrix effects were investigated using the post-extraction spiked method. The mean peak area (A) of BIT in the post-extracted spiked samples was compared with the corresponding peak area (B) obtained by directly injecting the standard analyte in the mobile phase at equivalent concentrations. All assays were repeated six times. The recovery and matrix effects of 10 ng/mL IS were evaluated using the same method. The assay precisions for the recovery and matrix effects were expected to be within ±15% RSD.

#### 3.6.6. BIT Stability

The stability of BIT in rat plasma or urine was evaluated by analyzing six replicates of plasma or urine samples at three QC levels (4, 400, and 1600 ng/mL) under the following storage conditions: (1) bench-top storage (6 h at room temperature), (2) long-term storage (2 months at –80 °C), (3) three freeze–thaw cycles, and (4) post-preparative/autosampler storage (24 h at 4 °C). All samples were analyzed using a calibration curve obtained from freshly spiked calibration standards and QC samples. Samples were considered stable if the percent deviation was within ±15.0% of the nominal concentration.

### 3.7. Application in BIT Pharmacokinetic Studies in Rats

#### 3.7.1. Intravenous Injection of BIT in Rats

The housing and handling of rats were performed using a previously reported method [21]. The jugular vein (for drug administration) and carotid arteries (for blood sampling) of each rat were cannulated using a polyethylene tube 50 (Clay Adams, Franklin Lakes, NJ, USA). Each rat was housed individually in a rat metabolic cage and allowed to recover from anesthesia for 4–5 h before starting the experiment. A dose of 10 mg/kg (1 mL/kg) BIT, dissolved in dimethyl sulfoxide, ethanol, and distilled water (10:15:75, *v/v/v*), was injected into the jugular vein of rats (*n* = 6 rats). Approximately 0.12 mL of blood from each rat was collected via the carotid artery at 0, 1, 5, 10, 15, 30, 45, 60, 90, 120, 180, and 240 min after intravenous injection. Heparinized 0.9% NaCl-injectable solution (20 units/mL, 0.3 mL) was used to flush the cannula immediately after each blood sampling to prevent blood clotting. Blood samples were immediately centrifuged at 18,000× *g* for 10 min at 4 °C, and plasma samples (50 μL) were stored at –80 °C until the LC–MS/MS analysis. Twenty-four hours after injection, each metabolic cage was rinsed with 10 mL of distilled water and the rinse was collected and combined with the 24 h urine samples. After measuring the exact volume of the combined urine samples, two 50 μL aliquots were stored at –80 °C until the LC–MS/MS analysis. At the same time (24 h after drug administration), each rat was euthanized with CO_2_ and the abdomen was opened. The entire gastrointestinal tract (including its contents and feces) was removed, transferred to a beaker containing 50 mL of methanol, and cut into small pieces using scissors. After sonication for 20 min, duplicate aliquots (50 μL) of the supernatant were collected from each beaker and stored at −80 °C until further use in the LC–MS/MS analysis.

#### 3.7.2. Tissue Distribution after Intravenous Injection of BIT in Rats

Following the plasma concentration–time profile after intravenous injection, 30 min and 180 min were selected as representative time points for the distribution phase and the elimination phase. In the tissue distribution study, BIT was dissolved in the same vehicle used in the intravenous study and administered to the rats at 10 mg/kg. Blood from each rat (n = 3 from each group) was collected from the carotid artery after 30 and 180 min, and each rat was euthanized using CO_2_ gas. After centrifugation of each blood sample, two 50 μL plasma aliquots were stored at −80 °C until the LC–MS/MS analysis. Following complete systemic perfusion with 0.9% injectable NaCl solution, approximately 0.5 g of brain, heart, kidney, liver, lung, and spleen samples from each rat (n = 3 from each group) were collected, washed with 0.9% injectable NaCl solution, and blotted dry with tissue paper. Each tissue sample was homogenized in 0.9% injectable NaCl solution at 1:2 (*w*/*v*) using a tissue homogenizer. Duplicate aliquots (50 μL) of the homogenates were collected and stored at −80 °C until further use in the LC–MS/MS analysis.

#### 3.7.3. Dermal Application

To examine the topical absorption of BIT, a reference sunscreen lotion formulation was modified according to the European Commission on Cosmetics and Medical Devices [22]. The lotion formulation weighed 40 g and consisted of two phases. Phase 1 comprised lanolin (4.8%), homosalate (7.7%), petrolatum (2.4%), stearic acid (3.8%), and propylparaben (0.048%). Phase 2 consisted of methylparaben (0.096%), disodium edetate (0.048%), propylene glycol (4.8%), triethanolamine (0.96%), and water (70.35%). The fatty phase 1 ingredients were melted and mixed at 80 °C. After complete solubilization, BIT was added to the phase 1 mixture, which was used as a 20-fold stock solution. Subsequently, phase 1 was added to aqueous phase 2 while stirring with a magnetic bar at 80 °C. Finally, the mixture was cooled to room temperature and stored in a glass bottle wrapped in aluminum foil. The freshly prepared lotion formulation was used in the pharmacokinetic analysis within 24 h.

The dermal application study was conducted using female rats in accordance with the Organization for Economic Cooperation and Development guidelines for in vivo skin absorption tests [23]. Twenty-four hours before the experiment, the female rats were anesthetized with isoflurane and their dorsal skin (8 × 11 cm^2^) was covered with shaving cream. After 5 min, the skin surface was shaved with an electric clipper and gently wiped with normal saline to remove fur debris. On the day of the experiment, the rats were randomly divided into three groups according to their treatment dose. The carotid arteries of the rats were cannulated for blood sampling and the rats were housed individually as described in Section 3.7.1. The lotion formulation was applied to a 7 × 10 cm^2^ area of shaved rat skin and covered with a dressing and bandage. The amount of dermally applied BIT lotion was 10 mg per rat (*n* = 8). Four hours after dermal application, the bandages were removed, and the exposed area was gently rinsed with normal saline solution to remove any lotion remaining on the skin. Additionally, the bandages and dressing were collected to estimate the amount absorbed through the skin during the 4 h application. The absorbed amount was estimated by subtracting the remaining and washing amount measured at the application site from the dermal application amount. Blood samples (0.12 mL) were collected from the carotid artery at 0, 90, and 240 min before and at 3, 5, 15, 30, 60, 120, 180, 240, and 1200 min after bandage removal. The blood samples were immediately centrifuged at 18,000× *g* for 5 min, and the plasma samples (50 μL) were stored at −80 °C until the LC–MS/MS analysis.

### 3.8. Pharmacokinetic Analysis

The following pharmacokinetic parameters for BIT were calculated via non-compartmental analysis using Phoenix^®^ WinNonlin^®^ (version 6.0; Certara USA, Princeton, NJ, USA): total area under the plasma concentration–time curve from time zero to time infinity (AUC_inf_) or to the last measured time t (AUC_t_), total body clearance (CL), terminal half-life (t_1/2_), mean residence time, apparent volume of distribution in a steady state (Vd_ss_), peak plasma concentration (*C*_max_), time to reach *C*_max_ (T_max_), and bioavailability (*F*) [16]. The dermal bioavailability (*F*) of BIT was calculated as follows:

*F* = (AUC_dermal/_AUC_iv_) × (dose_iv_/dose_dermal_) × 100

where AUC_dermal_ and AUC_iv_ are the AUC_inf_ values after the dermal and intravenous injection of BIT, respectively. All results were expressed as the mean ± standard deviation, except for T_max_, which was expressed as the median (range).

## 4. Conclusions

In this study, we developed and validated a simple and sensitive LC–MS/MS method for the quantification of BIT in various biological samples from rats. Our analytical LC–MS/MS method showed excellent performance, exhibiting advantages such as small plasma, urine, or tissue homogenate volume requirements for analysis (50 μL), high sensitivity (LLOQ: 2 ng/mL for plasma and urine, 10 ng/mL for tissue homogenates), short chromatographic run times (6 min), and a simple liquid–liquid extraction protocol. A pharmacokinetic study via intravenous and dermal routes was also conducted to investigate the pharmacokinetic properties of BIT. To the best of our knowledge, this was the first study to report BIT pharmacokinetics, including tissue distribution in rats. The findings of this study provide insights into the relationship between exposure and the toxic potential of BIT and a basis for future BIT risk assessment studies in humans.

## Figures and Tables

**Table 1:** Intra- and inter-day accuracy, precision, extraction recovery, and matrix effects of BIT in rat plasma and urine.

Matrices	Spiked Concentration (ng/mL)	Intra-Day (*n* = 6)		Inter-Day (*n* = 5)		Extraction Recovery Mean ± SD (%)	Matrix Effects Mean ± SD (%)
		Precision (RSD, %)	Accuracy (%)	Precision (RSD, %)	Accuracy (%)		
Plasma	2	11.3	100	8.66	104		
	4	4.31	99.4	4.33	101	90.8 ± 7.26	108 ± 3.58
	400	3.34	109	8.18	102	91.6 ± 6.00	107 ± 6.54
	1600	5.37	108	6.52	103	93.5 ± 6.25	102 ± 3.60
Urine	2	4.92	99.1	9.59	101		
	4	5.51	101	8.36	100	92.7 ± 2.72	100 ± 6.30
	400	3.96	95.6	4.95	103	91.3 ± 1.67	97.8 ± 3.48
	1600	4.39	108	3.82	109	90.4 ± 2.25	101 ± 4.66
IS	10					99.1 ± 4.54	102 ± 3.50

**Table 2:** Intra- and inter-day accuracy, precision, extraction recovery, and matrix effects of BIT in rat tissue homogenates.

Matrices	Spiked Concentration (ng/mL)	Intra-Day (*n* = 6)		Inter-Day (*n* = 5)		Extraction Recovery Mean ± SD (%)	Matrix Effects Mean ± SD (%)
		Precision (RSD, %)	Accuracy (%)	Precision (RSD, %)	Accuracy (%)		
Brain	10	12.8	96.4	12.9	96.3		
	30	5.65	99.8	11.4	100	86.6 ± 3.32	110 ± 3.58
	150	3.31	102	8.23	103	85.6 ± 4.43	103 ± 2.34
	800	6.08	105	5.38	107	90.3 ± 3.49	103 ± 8.44
Liver	10	7.25	100	10.2	101		
	30	5.40	95.7	4.92	106	85.8 ± 6.50	109 ± 3.08
	150	4.23	94.9	9.29	99.3	85.9 ± 5.65	105 ± 4.45
	800	5.26	101	8.34	103	86.5 ± 5.38	110 ± 3.66
Lung	10	8.64	99.4	7.12	107		
	30	3.72	102	9.65	104	87.8 ± 3.38	110 ± 5.61
	150	3.62	91.8	5.63	100	89.2 ± 3.02	105 ± 4.91
	800	7.24	97.7	4.92	106	84.4 ± 5.09	107 ± 6.33
Kidney	10	10.2	98.7	8.16	106		
	30	4.59	101	10.2	100	83.0 ± 4.32	103 ± 3.14
	150	5.15	98.8	6.45	106	86.2 ± 5.84	105 ± 5.81
	800	4.53	90.3	4.42	100	84.7 ± 3.31	107 ± 3.66
Heart	10	14.4	99.4	8.09	103		
	30	4.25	94.5	5.45	101	87.4 ± 4.14	103 ± 3.56
	150	5.40	94.2	6.60	96.2	84.5 ± 3.91	98.5 ± 8.34
	800	4.83	109	10.3	98.2	84.9 ± 5.35	104 ± 7.80
Spleen	10	13.7	98.0	12.3	96.5		
	30	8.96	103	5.08	95.5	86.7 ± 6.70	104 ± 5.49
	150	4.51	92.4	5.47	102	89.0 ± 3.83	102 ± 7.54
	800	5.96	105	10.4	99.4	93.3 ± 3.76	105 ± 5.08

**Table 3:** Stability of BIT in rat plasma and urine under various conditions (*n* = 6).

Storage Conditions	Concentration (ng/mL)		Precision (RSD, %)	Accuracy (%)
	Spiked	Measured (Mean ± SD)		
Plasma				
Bench-top stability	4	4.04 ± 0.230	5.76	101
(6 h at room temperature)	400	376 ± 22.8	5.70	93.9
	1600	1490 ± 74.1	4.63	92.8
Long-term stability	4	4.31 ± 0.182	4.55	108
(2 months at −80 °C)	400	448 ± 15.6	3.89	112
	1600	1720 ± 118	7.36	107
Freeze–thaw stability	4	3.77 ± 0.371	9.28	94.1
(three freeze–thaw cycles)	400	373 ± 21.7	5.41	93.2
	1600	1540 ± 58.8	3.68	95.9
Post-preparative/autosampler stability	4	3.99 ± 0.526	13.2	99.8
(24 h at 4 °C)	400	381 ± 24.2	6.05	95.3
	1600	1560 ± 98.0	6.13	97.6
Urine				
Bench-top stability	4	3.84 ± 0.288	7.52	95.9
(at room temperature for 6 h)	400	390 ± 19.0	4.87	97.5
	1600	1770 ± 23.4	1.32	111
Long-term stability	4	3.87 ± 0.264	6.83	96.8
(at −80 °C for 2 months)	400	412 ± 16.9	4.11	103
	1600	1720 ± 139	8.10	108
Freeze–thaw stability	4	4.04 ± 0.183	4.52	101
(three freeze–thaw cycles)	400	385 ± 25.9	6.74	96.2
	1600	1740 ± 46.6	2.68	108
Post-preparative/autosampler stability	4	3.84 ± 0.204	5.31	96.1
(at 4 °C for 24 h)	400	373 ± 21.1	5.65	93.2
	1600	1750 ± 44.7	2.56	109

**Table 4:** Pharmacokinetic parameters (mean ± SD) of benzisothiazolinone (BIT) after intravenous injection of 10 mg/kg BIT into rats.

Parameters (Units)	10 mg/kg (*n* = 6)
AUC_t_ (μg·min/mL) ^1^	359 ± 49.8
AUC_inf_ (μg·min/mL) ^2^	360 ± 49.9
t_1/2_ (min) ^3^	79.2 ± 10.4
CL (mL/min/kg) ^4^	28.3 ± 3.79
MRT (min) ^5^	4.60 ± 0.621
Vd_ss_ (mL/kg) ^6^	129 ± 19.0
Ae_0–24 h_ (% dose) ^7^	0.0506 ± 0.0136
GI_24 h_ (% dose) ^8^	0.0324 ± 0.00639

^1^ Total area under the plasma concentration–time curve from time zero to the last time t; ^2^ total area under the plasma concentration–time curve from time zero to infinity; ^3^ terminal half-life; ^4^ total body clearance; ^5^ mean residence time; ^6^ apparent volume of distribution at steady state; ^7^ the percentage of unmetabolized BIT excreted in urine over the course of 24 h; ^8^ the percentage of unmetabolized BIT recovered from the gastrointestinal tract, including its contents and feces at 24 h.

**Table 5:** Pharmacokinetic parameters (mean ± SD) of benzisothiazolinone (BIT) after the dermal application of BIT on rats.

Parameters (Units)	Dermal Application Amount
	10 mg/Rat (*n* = 8)
Dermal absorbed amount for 4 h (mg)	0.720 ± 0.122
AUC_t_ (μg·min/mL) ^1^	8.58 ± 0.905
AUC_inf_ (μg·min/mL) ^2^	10.6 ± 2.39
t_1/2_ (min) ^3^	101 ± 15.7
*C*_max_ (ng/mL) ^4^	41.1 ± 7.87
T_max_ (min) ^5^	245 (240–255)
*F* (%) ^6^	11.0

^1^ Total area under the plasma concentration–time curve from time zero to the last time t; ^2^ total area under the plasma concentration–time curve from time zero to infinity; ^3^ terminal half-life; ^4^ peak plasma concentration; ^5^ time to reach *C*_max_; ^6^ dermal bioavailability.

## Data Availability

Not applicable.

## Sample Availability

Samples of the compounds are not available from the authors.
